# Adenosine thiamine triphosphate accumulates in *Escherichia coli *cells in response to specific conditions of metabolic stress

**DOI:** 10.1186/1471-2180-10-148

**Published:** 2010-05-21

**Authors:** Tiziana Gigliobianco, Bernard Lakaye, Pierre Wins, Benaïssa El Moualij, Willy Zorzi, Lucien Bettendorff

**Affiliations:** 1 GIGA-Neurosciences, University of Liège, B-4000 Liège, Belgium; 2 Department of Human Histology-CRPP, University of Liège, B-4000 Liège, Belgium

## Abstract

**Background:**

*E. coli *cells are rich in thiamine, most of it in the form of the cofactor thiamine diphosphate (ThDP). Free ThDP is the precursor for two triphosphorylated derivatives, thiamine triphosphate (ThTP) and the newly discovered adenosine thiamine triphosphate (AThTP). While, ThTP accumulation requires oxidation of a carbon source, AThTP slowly accumulates in response to carbon starvation, reaching ~15% of total thiamine. Here, we address the question whether AThTP accumulation in *E. coli *is triggered by the absence of a carbon source in the medium, the resulting drop in energy charge or other forms of metabolic stress.

**Results:**

In minimal M9 medium, *E. coli *cells produce AThTP not only when energy substrates are lacking but also when their metabolization is inhibited. Thus AThTP accumulates in the presence of glucose, when glycolysis is blocked by iodoacetate, or in the presence lactate, when respiration is blocked by cyanide or anoxia. In both cases, ATP synthesis is impaired, but AThTP accumulation does not appear to be a direct consequence of reduced ATP levels. Indeed, in the CV2 *E. coli *strain (containing a thermolabile adenylate kinase), the ATP content is very low at 37°C, even in the presence of metabolizable substrates (glucose or lactate) and under these conditions, the cells produce ThTP but not AThTP. Furthermore, we show that ThTP inhibits AThTP accumulation. Therefore, we conclude that a low energy charge is not sufficient to trigger AThTP accumulation and the latter can only accumulate under conditions where no ThTP is synthesized. We further show that AThTP production can also be induced by the uncoupler CCCP but, unexpectedly, this requires the presence of pyruvate or a substrate yielding pyruvate (such a D-glucose or L-lactate). Under the conditions described, AThTP production is not different when RelA or SpoT mutants are used.

**Conclusions:**

In *E. coli*, AThTP accumulates in response to two different conditions of metabolic stress: lack of energy substrates (or inhibition of their metabolization) and uncoupled pyruvate oxidation. Both conditions prevent bacterial growth. There is no obvious link with the stringent response or catabolite repression.

## Background

Thiamine (vitamin B1) is an essential molecule for both prokaryotic and eukaryotic organisms, mainly because its diphosphorylated form (thiamine diphosphate, ThDP) is an indispensable cofactor for energy metabolism. In microorganisms, thiamine monophosphate (ThMP) is an intermediate in ThDP synthesis but, like free thiamine, it has no known physiological function. In addition to ThMP and ThDP, three other phosphorylated thiamine derivatives have been characterized: thiamine triphosphate (ThTP), and the newly discovered adenylated derivatives adenosine thiamine diphosphate (AThDP) [1] and adenosine thiamine triphosphate (AThTP) [1,2]. ThTP was discovered more than 50 years ago [3] and was found to exist in most organisms from bacteria to mammals [4]. Its biological function(s) remain unclear but, in *E. coli*, it was shown to accumulate transiently as a response to amino acid starvation, suggesting that it may be a signal required for rapid adaptation of the bacteria to this kind of nutritional downshift [5].

The recent discovery of adenylated thiamine derivatives has complicated the picture. First, these derivatives are unlikely to exert any cofactor role similar to the catalytic role of ThDP in decarboxylation reactions for instance. Indeed, the latter mechanisms rely on the relative lability of the C-2 proton of the thiamine moiety, evidenced by a chemical shift (9.55 ppm) definitely higher than expected for usual aromatic protons (7.5 - 8.5 ppm). In adenylated derivatives, the chemical shift of the C-2 proton is intermediate (9.14 - 9.18 ppm), suggesting a through-space interaction between thiazole and adenylyl moieties, and a U-shaped conformation of these molecules in solution [1]. This is not in favor of a possible catalytic cofactor role of AThDP or AThTP, which are more likely to act as cellular signals.

AThDP has been only occasionally detected in biological systems (and only in very low amounts), but AThTP, like ThTP, can be produced by bacteria in appreciable quantities (~15% of total thiamine) under special conditions of nutritional downshift: while ThTP accumulation requires the presence of a carbon source such as glucose or pyruvate [5], accumulation of AThTP is observed as a response to carbon starvation [2]. In *E. coli*, the two compounds do not accumulate together: their production indeed appears as a response to specific and different conditions of metabolic stress.

Little is known about the biochemical mechanisms underlying the synthesis and degradation of triphosphorylated thiamine derivatives. No specific soluble enzyme catalyzing ThTP synthesis was characterized so far. In contrast, a soluble enzyme preparation catalyzing AThTP synthesis from ThDP + ADP or ThDP + ATP was obtained from *E. coli *extracts. This hypothetical ThDP adenylyl transferase could be partially characterized, but its catalytic efficiency seems rather low and the protein, that appears to be a high molecular mass complex, could not be obtained in pure form. The observation that both ADP and ATP are substrates for the reaction may seem surprising, as it might be expected that AThTP synthesis, as a response to the energy stress caused by carbon starvation, should be activated when the [ADP]/[ATP] ratio is high and inhibited when it is low. Most probably, other unidentified factors are important for controlling the rates of synthesis and degradation of AThTP. The present study is a first attempt to delineate the exact conditions and mechanisms leading to AThTP production in *E. coli*. We show that there is no direct relationship between this response and a low cellular ATP content. Unexpectedly, we find that the proton motive force is also an essential factor controlling AThTP production. Finally, the possible relationships with the stringent response are examined.

## Results and Discussion

### *E. coli *cells slowly accumulate AThTP in response to carbon starvation

**Figure 1:**
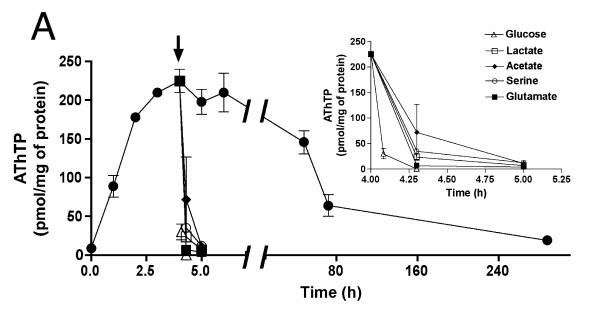
**AThTP levels as a function of time in BL21 cells transferred to minimal medium**. (A) The bacteria were grown overnight in LB medium, transferred to M9 minimal medium and incubated at 37°C at 250 rpm in the absence of a carbon source. At the time indicated, 1 mL aliquots were taken for determination of thiamine derivatives. The arrow in (A) indicates the addition of either 10 mM D-glucose, L-lactate, acetate, L-serine or L-glutamate. The inset shows the decrease of AThTP levels on an expanded time scale. (Means ± SD, n = 3)

*E. coli *cells have a high total thiamine content (~1 nmol/mg of protein). Under optimal conditions of growth (in LB medium), thiamine exists mainly as ThDP (> 95% of total thiamine) and ThMP (3-4%). ThTP and AThTP are found only in traces. We have previously shown that when the bacteria are transferred to a minimal M9 medium devoid of any carbon source, AThTP starts to accumulate and a maximum (about 15% of total thiamine) is reached after 4 hours. Here, we show that AThTP levels could be maintained for two days (Figure 1A) suggesting that most cells survive during this period. Then, the AThTP content gradually decreased, but this was probably due to death of the bacteria: indeed, the ability to form colonies after plating on agar plates decreased and became null after 6 days (data not shown), a test generally used to determine bacterial survival [6]. Luo et al. [7] reported that after two days of glucose starvation, about 54% of BL21 cells survived aerobically, which is in agreement with the present data.

We attempted to analyze the possible relationship between the appearance of AThTP and the decrease in ATP levels caused by carbon starvation. When the cells were transferred to M9 medium after growth in LB medium, their ATP content decreased from 5.8 to 1.6 nmol per mg of protein. This corresponds to a decrease in intracellular concentration from 1.8 to 0.5 mM, assuming an intracellular volume of 3.2 mL/mg of protein, [8]). The drop (about 70%) was rapid, occurring in less than 30 min, but the subsequent decrease in ATP levels was slow, the intracellular concentration after several hours remaining ≥ 0.3 mM in spite of the absence of a carbon source. This suggests that the bacteria are able to use endogenous energy sources (such as glycogen for instance) in order to maintain a minimal energy charge, allowing survival, but not growth.

When AThTP was allowed to accumulate for 4 h in the absence of a carbon source, addition of various metabolizable substrates induced a sharp decrease in AThTP content (inset of Figure 1). As previously shown [2], glucose addition (10 mM) triggered a drop of 80-90% in AThTP in less than 5 min and nearly 100% after 30 min, while the decrease was slower with other carbon sources (especially succinate and acetate).

We also confirmed that virtually no AThTP was produced when a metabolizable carbon source was present at zero time (when bacteria were transferred from LB to M9 medium). As shown in Table 1, glucose was very effective in antagonizing AThTP accumulation, as an external concentration as low as 1 mM reduced the AThTP content (measured after 60 min) by about 80% while a concentration ≥ 5 mM nearly completely prevented the accumulation of AThTP. However, at high ionic strength (1 M NaCl, KCl or choline chloride), glucose was unable to prevent AThTP accumulation. This is not surprising, as the high ionic strength is known to impair glucose utilization by *E. coli *cells [9].

**Table 1:** Effect of various carbon sources on AThTP production in the BL21 *E. coli *strain.

	AThTP(pmol/mg of protein)
Control	88 ± 6
D-Glucose (1 mM)	13 ± 4
D-glucose (2.5 mM)	9 ± 2
D-Glucose (5 mM)	< 2
D-Glucose (10 mM)	< 2
L-Lactate (10 mM)	14 ± 2
Succinate (10 mM)	6 ± 1
L-Malate (10 mM)	8 ± 2
D-Glucose (10 mM) + NaCl (1.2 M)	94 ± 13
D-Glucose (10 mM) + KCl (1.2 M)	92 ± 6
D-Glucose (10 mM) + Choline Cl (1.2 M)	131 ± 15
Streptomycin^a ^(10 μM)	62 ± 2
Neomycin^a ^(10 μM)	68 ± 3
AA^b^	12 ± 2
AA^b ^+ serine hydroxamate (0.5 mg/mL)	18 ± 2

^a^All amino acids (40 μg/mL each) with the exception of serine

^b^No carbon source present

The bacteria (A_600 _> 1) were incubated for 60 min at 37°C in minimal M9 medium containing substrates at the concentrations indicated. Mean ± SD for 3 - 9 experiments.

**Figure 2:**
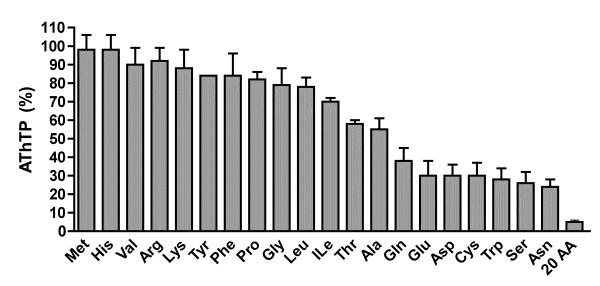
**Effect of amino acids on the accumulation of AThTP in minimum medium**. The bacteria were incubated for 30 min in M9 medium (in the absence of glucose) and in the presence of amino acids (10 mM each, except for Tyr which was at 5 mM). The amino acid mixture (20 AA) contained all amino acids at a concentration of 0.5 mM, except for tyrosine (0.05 mM) and tryptophan (0.1 mM). The results are expressed as percentage of AThTP appearing in 30 min in the absence of any carbon source. (Means ± SD, n = 3).

The antibiotics streptomycin and neomycin have little effect on AThTP accumulation in the absence of a carbon source, suggesting that protein synthesis is not required for AThTP accumulation. We also wanted to know whether the appearance of AThTP was specifically linked to carbon starvation or could be triggered by other forms of nutritional downshifts or cellular stress. As reported earlier [2], there was no AThTP production in response to phosphate or nitrogen starvation when a carbon source was present. However, as shown in Figure 2, some amino acids can prevent AThTP accumulation (in the absence of glycolytic or Krebs cycle substrates) presumably because they can be used as carbon (and energy) sources. Indeed, amino acids that are rapidly degraded (such as serine, glutamine, glutamate and aspartate) are the most efficient.

Finally, it should be stressed that AThTP could never be detected in appreciable amounts in exponentially growing bacteria: its appearance was always associated with a downshift of growth. However, the onset of the stationary phase at the end of exponential growth did not result in accumulation of AThTP (data not shown). This suggests that the appearance of this compound is essentially a response of the bacteria to a sudden nutritional downshift (carbon starvation) or other forms of energy stress (see below) but it does not seem to play a role in stationary phase physiology.

### AThTP synthesis is unrelated to the stringent response and polyphosphate production

It is well known that amino acid starvation induces the so-called stringent response [10] to nutritional downshifts. When the bacteria are transferred to minimal medium containing no amino acids, (p)ppGpp rapidly accumulates, reaching a maximum value in one minute or less. This response can also be induced in the presence of a mixture of amino acids where serine is replaced by serine-hydroxamate [11]. When the bacteria (BL21 strain) were incubated in M9 medium under these conditions (all amino acids, except serine, present at a concentration of 40 μg/mL and serine-hydroxamate, 0.5 mg/mL), AThTP levels remained low (Table 1). Further evidence that the stringent response is not directly implicated in the production of AThTP is provided by the use of mutants defective in enzymes responsible for the synthesis of (p)ppGpp. Indeed, bacteria devoid of RelA activity, a ribosome-associated enzyme catalyzing the synthesis of (p)ppGpp activated during amino acid starvation [10], produce normal amounts of AThTP during carbon starvation (Table 2). Furthermore, we tested a strain deficient in SpoT [12], a bifunctional enzyme having both (p)ppGpp hydrolyzing and synthesizing activity. This protein is probably involved in fatty acid starvation sensing via the acyl carrier protein, leading to a switch from (p)ppGpp degradation to (p)ppGpp synthesis [13,14]. Like the BL21 strain, SpoT-deficient bacteria produced AThTP in minimal medium devoid of a carbon source (Table 2). These results suggest that production of (p)ppGpp is not a requirement for AThTP synthesis, and that we are dealing with a phenomenon that is unrelated to the stringent response.

**Table 2:** Effect of various carbon sources on AThTP production by different *E. coli *strains.

	AThTP (pmol/mg of protein)
**MG1655**	
Control	62 ± 6
D-Glucose (10 mM)	11 ± 2
L-Lactate (10 mM)	26 ± 8
Pyruvate (10 mM)	< 2
	
**RelA**^-^	
Control	56 ± 12
D-glucose	< 2
	
**SpoT**^-^	
Control	80 ± 6
D-Glucose	10 ± 3
	
**CF5802**	
Control	62 ± 4
D-Glucose (10 mM)	< 2
	
**CV2**	
Control	120 ± 11
D-glucose	< 2
L-lactate	< 2

^a^n. d., not determined

The bacteria (A_600 _> 1) were incubated for 20 min at 37°C in minimal M9 medium containing substrates at the concentrations indicated. Mean ± SD for 3 - 9 experiments.

The BL21 strain is particular in the sense that it lacks Lon protease, a protein important in the physiological response of bacteria to amino acid starvation [15]. During amino acid starvation, *E. coli *cells accumulate inorganic polyphosphate (poly-P) that activate Lon and redirect their activity towards free ribosomal proteins [16]. Whilst the survival rate of wild-type and Lon-deficient *E. coli *is the same under aerobic conditions, Lon-deficient cells are more sensitive to anaerobic conditions [7]. The degradation of these proteins releases amino acids that can be used to make enzymes required for amino acid metabolism [17]. In our experiments, the wild-type MG1655 strain largely behaved in the same way as the BL21 strain in accumulating AThTP in response to carbon starvation (Table 2). Furthermore, the CF5802 (MG1655 Δppk1-ppx) strain, deficient in polyphosphate kinase and exopolyphosphatase, and therefore unable to synthesize polyphosphate, also produced normal levels of AThTP during carbon starvation (Table 2).

### AThTP synthesis is triggered by metabolic inhibition

**Figure 3:**
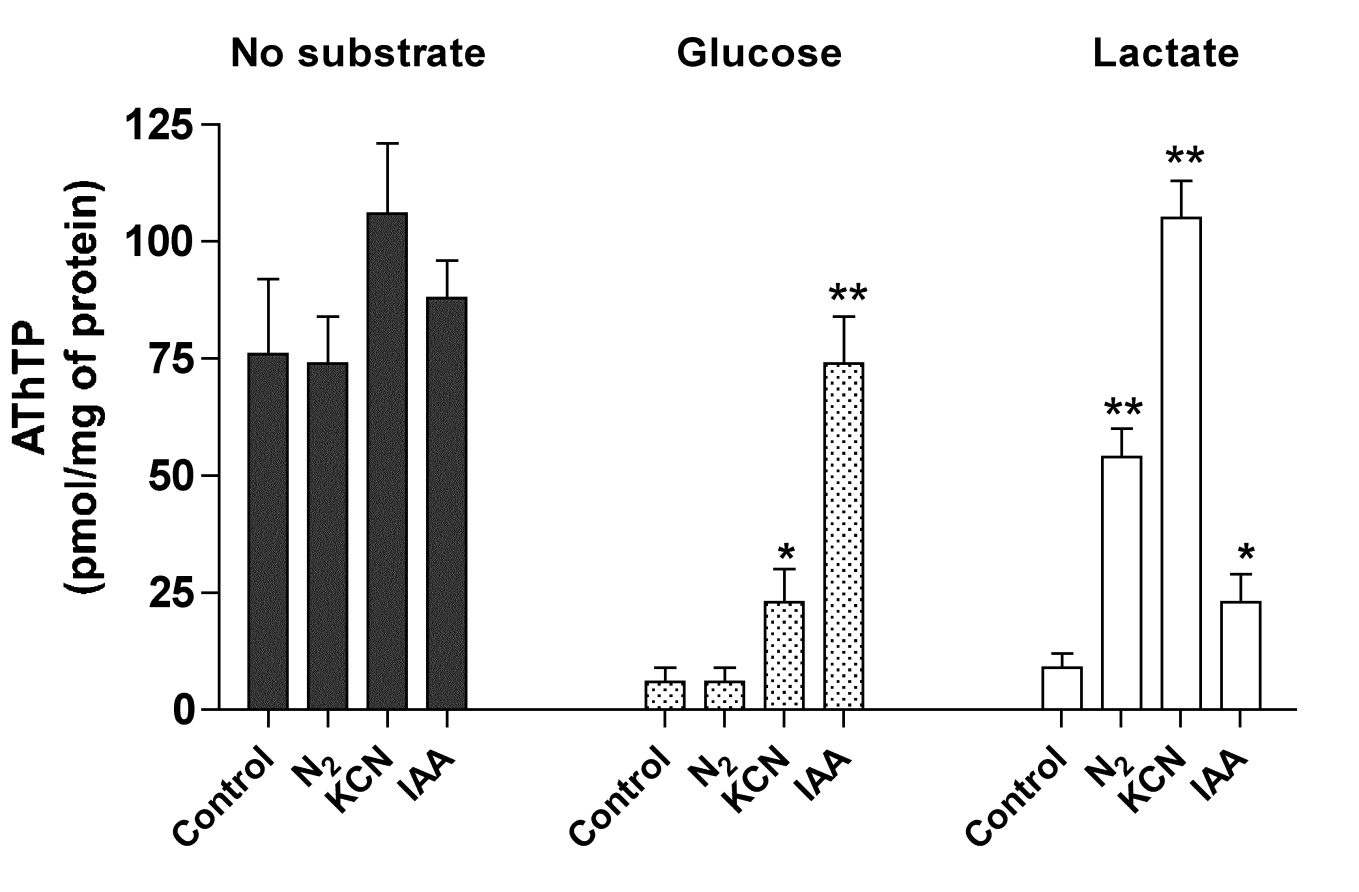
**Effect of metabolic inhibitors and anoxia on AThTP levels in BL21 cells**. The bacteria were grown overnight in LB medium and transferred to minimal medium in the absence or the presence of O_2 _(replaced by N_2_), KCN (1 mM) or iodoacetate (1 mM) (20 min, 37°C) either in the absence of substrates or in the presence of 10 mM D-glucose or 10 mM L-lactate. (**, p < 0.01; *, p < 0.05: two-way ANOVA followed by the Dunnett test for comparisons with the respective control. (Means ± SD, n = 4)

**Figure 4:**
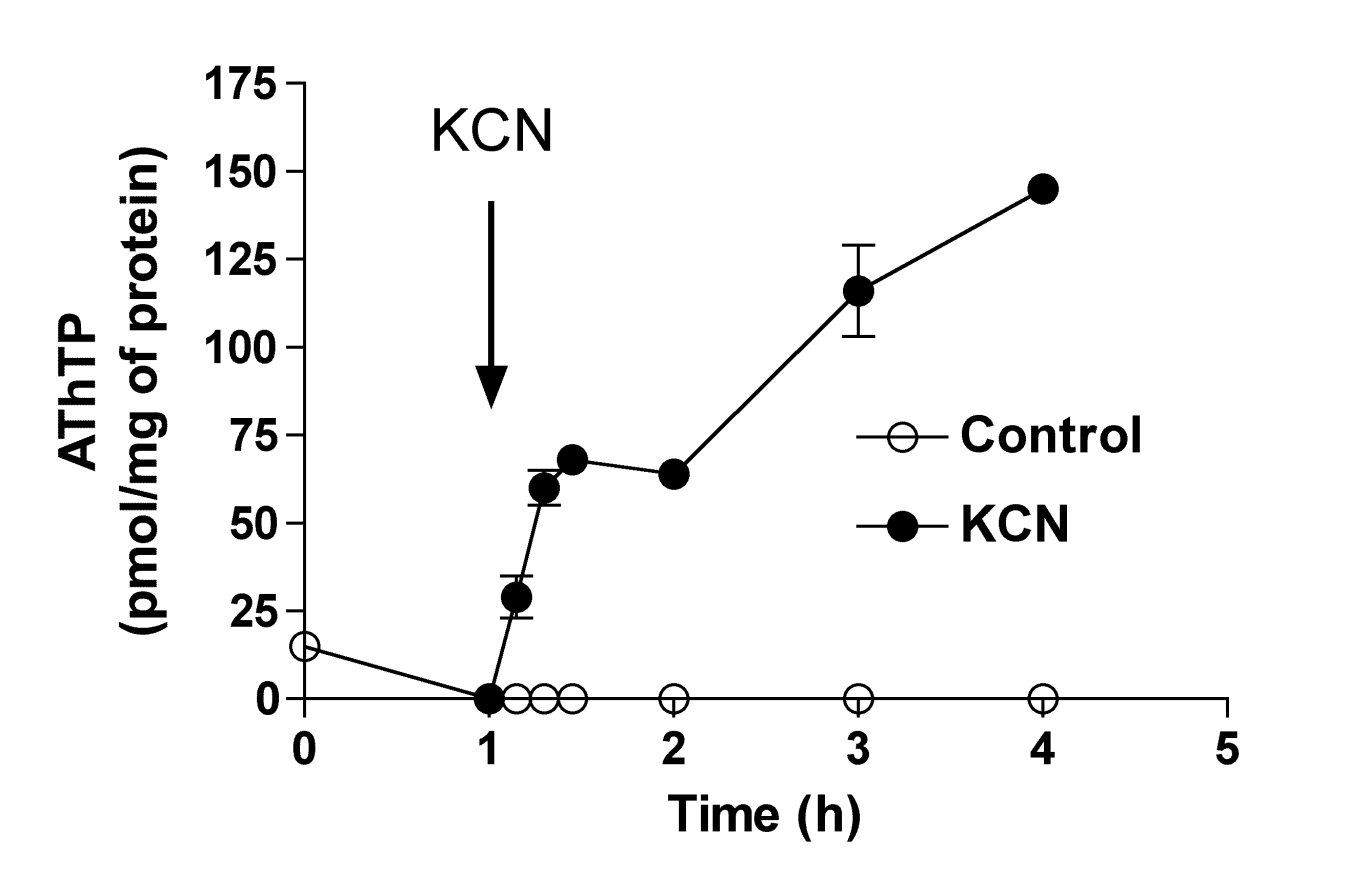
**Effect of KCN on AThTP levels in BL21 cells**. The bacteria (BL 21 strain) were grown overnight in LB medium, and transferred to M9 minimal medium and incubated at 37°C in the presence of 10 mM L-lactate. After 60 min, 1 mM KCN was added. (Means ± SD for 3 experiments)

We studied the effects of two metabolic inhibitors, iodoacetate and KCN in the presence of either D-glucose or L-lactate (Figure 3). With iodoacetate, an inhibitor of the glycolytic enzyme glyceraldehyde phosphate dehydrogenase [18], AThTP accumulated in the presence of glucose, but much less in the presence of lactate. However, the reverse was observed with KCN, an inhibitor of the respiratory chain. This is confirmed by data illustrated in Figure 4. In the presence of glucose, KCN induced a significant increase in AThTP levels; while in the presence of lactate, AThTP was strongly increased in the presence of KCN and during anoxia. This may be explained if, in the presence of glucose, glycolytic ATP can still be produced. These results demonstrate that, while AThTP accumulation can be induced by carbon starvation, it is also observed in the presence of a carbon source if the metabolization of the substrate is blocked. This would suggest that AThTP is produced when ATP production is inhibited, but further data show that AThTP accumulation is not directly linked to lowering of the energy charge (see below).

### Uncoupling of oxidative phosphorylation in the presence of a substrate induces a rapid accumulation of AThTP

**Figure 5:**
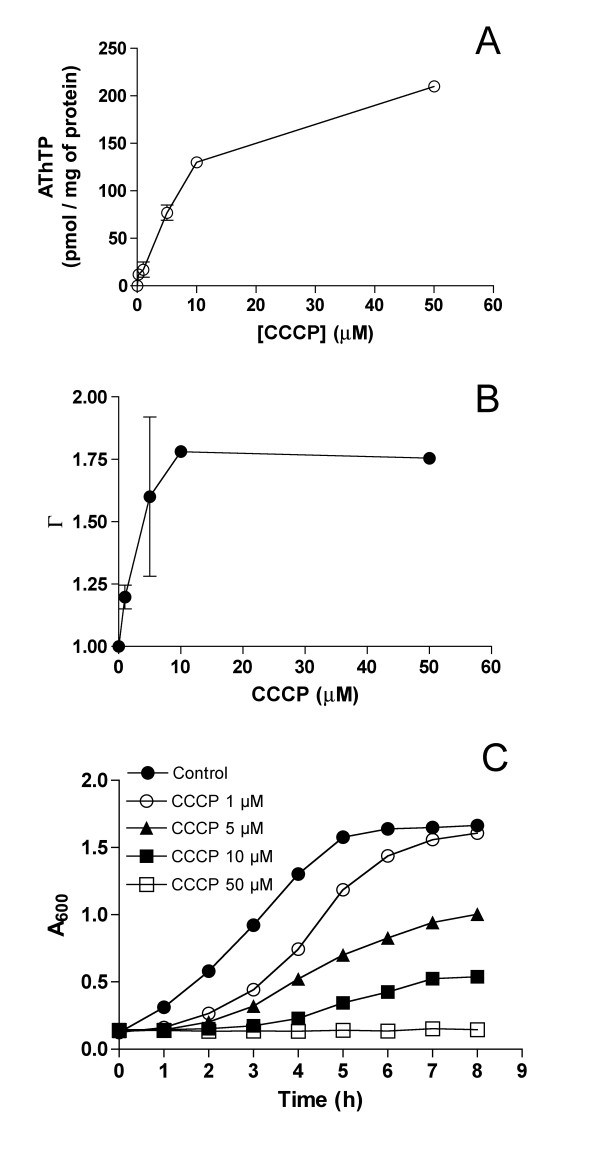
**Dose-dependent effects of CCCP on AThTP content, respiration and growth of *E. coli***. (A) The bacteria (BL21 strain) were transferred to minimal M9 medium containing 10 mM D-glucose and the indicated CCCP concentrations. After 20 min (37°C, 250 rpm), the intracellular AThTP concentration was determined by HPLC. (B) Effect of CCCP on the respiratory ratio Γ (O_2 _consumption in the presence of CCCP over the O_2 _consumption in the absence of CCCP) measured in the presence of 10 mM glucose at 37°C by polarographic recording of O_2 _consumption. (C) Growth curves of the bacteria in the presence various concentrations of CCCP. (Means ± SD, n = 3)

The most dramatic effect on AThTP levels was obtained in the presence of the uncoupler CCCP, which induced a rapid appearance of AThTP. *E. coli *cells (BL21 strain) were incubated for 20 min in the presence of glucose (10 mM) and increasing concentrations of CCCP (Figure 5A). The amount of AThTP increased with increasing concentrations of CCCP. This increase was paralleled by a stimulation of O_2 _consumption (Figure 5B). Progressive increase in CCCP concentration also led to an increased lag before the growth resumed (Figure 5C). The recovery of growth in the presence of low (< 10 μM) concentration of CCCP may be related to development by the bacteria of mechanisms of CCCP ejection [19]. In any event, the recovery was only partial in the presence of 5 or 10 μM CCCP and completely blocked at higher concentrations. These results suggest that the collapse of Δp favors the appearance of AThTP.

### A low energy charge is not sufficient to trigger AThTP accumulation

Our results indicate that carbon starvation is a robust trigger of AThTP accumulation in *E. coli *cells, whatever the strain used (see Table 2). However, AThTP can also be produced in the presence of a carbon source when metabolic inhibitors are present, suggesting that AThTP production is linked to metabolic inhibition and/or energy stress rather than the absence of an extracellular carbon source. An alternative possibility is that AThTP accumulation might be triggered by dissipation of the Δp rather than by a drop of energy charge.

A useful tool for answering those questions is the thermo-sensitive CV2 strain [20,21]. This strain contains a heat-sensitive AK that is rapidly inactivated when the bacteria are grown at temperatures higher than 30°C. At 37°C, the cellular energy charge drops within two hours from 0.9 to 0.2, the intracellular ATP concentration being around 0.2-0.3 mM. When an energy substrate is present, ATP is produced at a normal rate, but its hydrolysis coupled to nucleic acid synthesis results in an accumulation of AMP that cannot be converted to ADP because of lack of AK activity. Therefore, the energy charge remains low despite the presence of an energy substrate. Here, we observe that at 37°C, CV2 cells accumulate AThTP in the absence of carbon sources as expected, but not when D-glucose or L-lactate are present (Table 2). This is surprising, as the presence of those substrates does not induce any substantial increase in intracellular ATP concentration. Thus, AThTP production does not occur in the presence of substrates, even when the energy charge remains very low. However, under these conditions ThTP levels are very high [21] and it is therefore possible that AThTP accumulation is inhibited by ThTP (see below).

**Figure 6:**
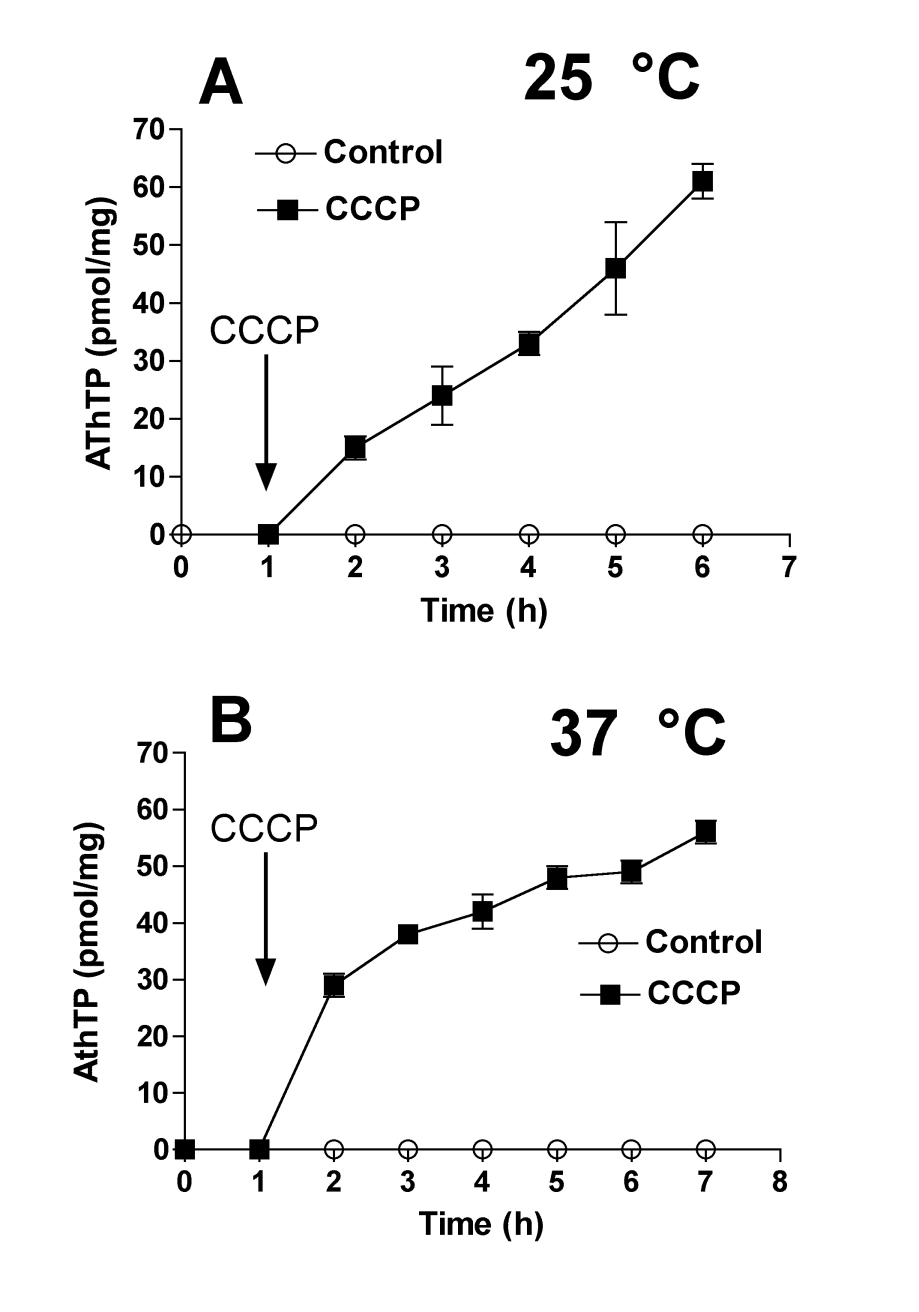
**Effect of CCCP on AThTP levels in the *E. coli *CV2 strain incubated in minimal medium containing L-lactate at 25 and 37°C**. The bacteria were grown overnight in LB medium and transferred to minimal M9 medium containing 10 mM L-lactate either at 25 or at 37°C. CCCP (50 μM) was added after 60 min (arrow). (Means ± SD, n = 3)

The effects of the uncoupler CCCP were also investigated in CV2 cells. The cells were transferred to a minimal medium supplemented with L-lactate (10 mM) either at 25°C (Figure 6A) or at 37°C (Figure 6B) and CCCP was added after 1 hour. At 25°C addition of CCCP induced a rapid decrease of the energy charge (from 0.9 ± 0.1 to 0.3 ± 0.1 after 20 min). In contrast, at 37°C, addition of CCCP only slightly decreased the energy charge as it was already very low (from 0.29 ± 0.04 to 0.26 ± 0.02 after 20 min and less than 0.2 after 1 h). However, at both temperatures, CCCP induced a rapid increase in AThTP content. This change occurred even more rapidly at 37°C than at 25°C. At 37°C, ATP content was less than 1 nmol per mg protein (corresponding to an intracellular concentration of 0.3 mM) 1 h after addition of CCCP. Thus AThTP accumulation occurred when the Δp was abolished and did not appear to be significantly influenced by variations in the ATP pool.

At both temperatures, CCCP increased the respiratory rate by a factor of approximately 2 with glucose (from 21 ± 7 to 41 ± 9 nmol.mg^-1^.min^-1^, n = 3) and L-lactate (from 19 ± 8 to 38 ± 1 nmol.mg^-1^.min^-1^, n = 3) as substrates. These results suggest that the CV2 strain retains a significant Δp even at 37°C, when the energy charge is very low.

The maintenance of this proton motive force is linked to proton pumping by the respiratory chain and requires the presence of an energy substrate and oxygen. Under these conditions, CCCP triggers AThTP production presumably by collapsing Δp. This is observed at 37°C as well as at 25°C. At 37°C, CCCP does not substantially affect the energy charge. Therefore, our results with the CV2 strain strongly suggest that Δp is more important than the energy charge as a factor controlling AThTP production.

**Figure 7:**
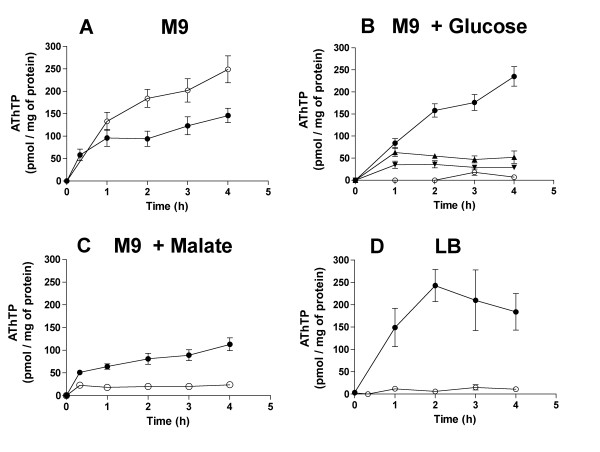
**Effect of CCCP on the AThTP content of BL21 cells in minimal M9 medium**. The bacteria were grown overnight in LB medium and then transferred to M9 minimal medium at 37°C in the absence of substrate (A) or in the presence of 10 mM D-glucose (B), L-malate (C) or in LB medium (D) with (●) or without (○) CCCP (50 μM). In B, iodoacetate was present at 1 (▲) and 5 (▼) mM final concentration. (Means ± SD, n = 3)

Further investigations showed, however, that factors other than Δp are also important for the control of intracellular AThTP levels. Indeed, when AThTP accumulates under carbon starvation, this accumulation is not accelerated by CCCP. Actually, we consistently found that under these conditions CCCP had a negative effect on AThTP accumulation (Figure 7A). However, CCCP induced a greater accumulation of AThTP in the presence of glucose (Figure 7B).

Furthermore, the activating effect of glucose was counteracted by iodoacetate, suggesting that the activation is induced by a degradation product rather than by glucose itself. On the other hand, we found that L-malate was much less effective than glucose as an activator of AThTP production in the presence of CCCP (Figure 7C). A good effect of CCCP was also obtained in LB medium (Figure 7D), probably because of the presence of amino acids entering the glycolytic pathway. This suggests that the unidentified activator can be produced by glucose but not by malate oxidation. It is interesting to point out that the enzyme catalyzing AThTP synthesis in vitro is also activated by an unidentified heat-stable factor [4].

### ThTP inhibits the accumulation of AThTP

**Figure 8:**
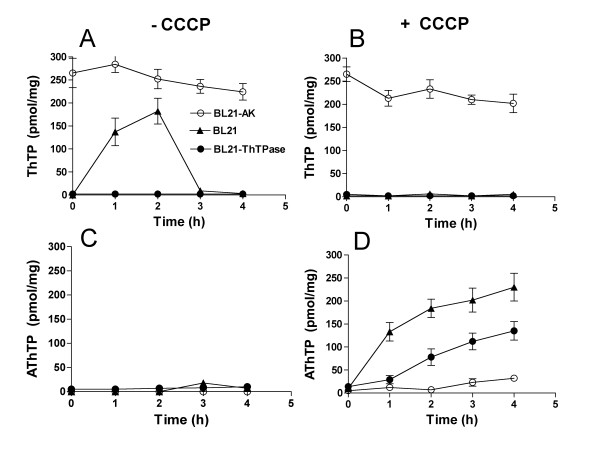
**Effect of intracellular ThTP levels on AThTP accumulation**. BL21 strains overexpressing *E. coli *AK (○) or GST-hThTPase (●) were grown overnight in LB medium containing ampicillin (0.1 mg/mL). The cultures were diluted to a density of A_600 _= 0.6 - 0.8 and protein expression was induced with IPTG (1 mM) for 3 h. Then the bacteria were transferred to a minimal medium containing 10 mM glucose without (A, C) or with CCCP 50 μM (B, D) and ThTP and AThTP were determined as a function of time. For comparison an experiment with the control BL21 strain (▲) is also shown. (Means ± SD, n = 3)

As ThTP and AThTP accumulate under different conditions and AThTP is never observed in the presence of ThTP, we wondered whether ThTP might inhibit the accumulation of AThTP. In order to check this possibility, we used BL21 strains overexpressing either *E. coli *AK or GST-hThTPase (a highly specific recombinant human ThTPase). When highly overexpressed in BL21 cells, bacterial AK catalyzes ThTP synthesis [21], leading to an accumulation of high amounts of ThTP (about 10 - 15% of total thiamine), whatever the composition of the medium (presence of glucose or not). Overexpression of AK leads to approximately a 1000-fold increase in AK protein compared to endogenous AK. GST-hThTPase is a highly specific and efficient enzyme that hydrolyzes all intracellular ThTP and when it is overexpressed, the cells are unable to accumulate significant amounts of ThTP [5]. Both enzymes were overexpressed for 3 h in the presence of IPTG and then the bacteria were transferred to a M9 medium containing glucose with or without 50 μM CCCP (Figure 8). In the BL21-AK strain, ThTP levels remained high for several hours, while no ThTP was observed in the BL21-hThTPase strain (Figure 8A). For comparison, the behavior of a normal BL21 strain is also shown. Under these conditions, no significant amount of AThTP was observed in any of the three strains (Figure 8C). However, AThTP levels increased much more rapidly in the BL21-hThTPase strain than in the BL21-AK strain (Figure 8D), suggesting that there is indeed an inhibitory effect of ThTP on AThTP accumulation.

### Mechanism of AThTP synthesis

**Figure 9:**
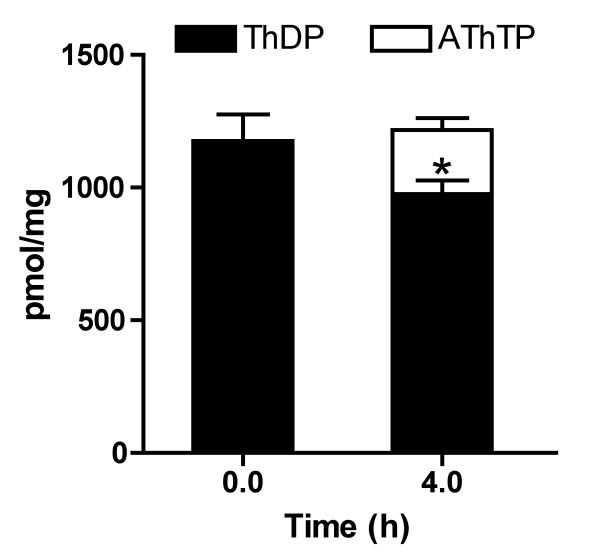
**AThTP is formed from ThDP**. The bacteria were incubated in minimal M9 medium and thiamine derivatives were determined at zero time and after incubation for 4 h. The results are expressed as mean ± SD for 3 experiments (*, p < 0.05; one-way ANOVA followed by the Dunnett post-test for comparison with ThDP levels at t = 0).

In the absence of substrates, accumulation of AThTP was concomitant with a decrease in cellular ThDP, while the total thiamine content (ThDP +AThTP) remained constant (Figure 9). These results show that part of the intracellular ThDP can be converted to AThTP. Indeed, we previously showed that AThTP can be formed enzymatically according to the reaction ThDP + ADP (ATP) ⇆ AThTP + P_i _(PP_i_) [22]. Both ATP and ADP can be the phosphate donor for this reaction but the fact that AThTP is synthesized under conditions where ATP are low (see Table 1) suggests that the physiological phosphate donor for the above reaction is ADP rather than ATP.

We determined the intracellular proportions of free *vs *protein-bound ThDP after fractionation on a molecular sieve (TSK gel column). Most of the ThDP in the supernatant was eluted in the inclusion volume of the column. Only about 15 ± 4% of the ThDP was eluted in the void volume, associated with the high-molecular weight protein fraction. As ThDP is generally rather tightly bound to its apoenzymes, this result suggests that most of the cellular ThDP corresponds to a free pool (intracellular concentration of about 250 μM). All AThTP was eluted in the inclusion volume, suggesting that it is essentially free in the cytosol, or at least not tightly bound to proteins.

Therefore, the pool of free ThDP in *E. coli *appears to be a reservoir for the production of triphosphate compounds under certain conditions of stress. This does not exclude that free ThDP might have other physiological roles.

## Conclusion

In *E. coli*, AThTP can be synthesized from free cellular ThDP and ADP or ATP. It accumulates (up to 15% of total thiamine) in response to different conditions of metabolic stress that impair bacterial growth: carbon starvation, metabolic inhibition or dissipation of the electrochemical proton gradient. These conditions are associated with different degrees of energy failure, but there is no direct relationship between AThTP production and decreased intracellular ATP levels. It might be argued that AThTP is a kind of ATP storage form. This is however unlikely as the maximum concentrations attained are two orders of magnitude lower than ATP concentrations. Furthermore, hydrolysis of AThTP yields ThDP and therefore, the other product of hydrolysis must be AMP and not ATP.

Our results show that AThTP accumulation is inhibited by high intracellular concentrations of ThTP. This may explain at least in part, that the two compounds never accumulate together in *E. coli *cells.

It is finally demonstrated that glucose and other substrates yielding pyruvate are very effective to induce the fast disappearance of AThTP after prolonged incubation of the cells in the absence of a carbon source. Surprisingly, the same substrates also enhance the appearance of AThTP when the proton motive force is abolished. Those data suggest that intracellular AThTP levels are regulated by multiple factors, including the electrochemical proton gradient, the intracellular concentration of ThTP and an unidentified factor whose synthesis is linked to pyruvate oxidation.

With this respect it is noteworthy that there is an important accumulation of cAMP during carbon starvation in *E. coli *due to the stimulation of adenylate cyclase. The regulation of this enzyme is dependent on substrate uptake systems, but not on Δp or decreased ATP levels [23]. Furthermore, uncouplers such as DNP or CCCP decrease adenylate cyclase activity, suggesting that the well-known catabolite repression in *E. coli *is not involved in increased AThTP levels during carbon starvation. The fact that *E. coli *strains deficient in RelA and SpoT activity normally synthesize AThTP suggests that (p)ppGpp and the stringent response are not involved AThTP synthesis. This hypothesis is further supported by the absence of effect of serine hydroxamate on its accumulation.

AThTP is never observed in growing bacteria, or under conditions where ATP levels are high. This, suggests that AThTP might be a factor involved in the adaptation of the bacteria to conditions of energy stress. However, a low energy charge does only lead to AThTP accumulation under conditions where ThTP is absent.

## Methods

### Chemicals

All chemicals were either from Sigma-Aldrich NV/SA (Bornem, Belgium) or from Merck (Darmstadt, Germany) and of the highest purity available. ThTP and AThTP were prepared as described [1,24].

### *E. coli *strains

The BL21 strain, lacking OmpT and Lon proteases, was from Amersham Biosciences. The MG1655 (wild-type K-12) strain and the CF5802 strain [25], deficient in polyphosphate kinase and exopolyphosphatase (MG1655 Δ*ppk-ppx::km*) were gifts from Dr. M. Cashel (Laboratory of Molecular Genetics, NICHD, National Institutes of Health, Bethesda, MD, USA). The heat-sensitive CV2 (CGSC # 4682, initially derived from *E. coli *strain K-10) [26] and the SpoT-deficient NF161 (CGSC # 5244 derived from K-12) [12] strains were obtained from the *E. coli *Genetic Resource Center (Yale University, New Haven, CT, USA). A strain devoid of RelA (MFT702 ΔrelA derived from MG1655) [10] was a gift from Dr. T. Conway (Advanced Center for Genome Technology, University of Oklahoma, Norman, OK, USA). The BL21 strains overexpressing either human recombinant ThTPase as GST fusion protein (BL21-hThTPase) or *E. coli *adenylate kinase (BL21-AK) were produced as previously described [21,27].

### Growth and processing of the bacteria

The bacteria were grown overnight (37°C, 250 rpm) in 50-100 mL Luria-Bertani (LB) medium (tryptone, 10 g/L; yeast extract, 5 g/L; NaCl, 10 g/L, pH 7.0). The bacteria were centrifuged (5 min; 5000 × g) and suspended in the initial volume of M9 minimal medium (Na_2_HPO_4_, 6 g/L; KH_2_PO_4_, 3 g/L; NaCl, 0.5 g/L; NH_4_Cl, 1 g/L; CaCl_2_, 3 mg/L; MgSO_4_, 1 mM, pH 7.0) containing various metabolic substrates in sterile PS-tubes (18,0/95 mm, 14 mL, Greiner Bio-One BVBA/SPRL, Wemmel, Belgium). If not otherwise stated, the bacteria were incubated at 37°C with shaking (250 rpm). The density of the cultures was determined by reading the absorbance at 600 nm (A_600_). After incubation, the bacteria were sedimented as above, the pellets were suspended in 12% TCA, the precipitated proteins were spun down (15 min, 15,000 × g) and the pellet was dissolved in 0.8 N NaOH for protein determination by the method of Peterson [28]. The supernatant was treated with diethyl ether to remove TCA and analyzed by HPLC for thiamine compounds [29]. For the determination of adenine nucleotides by HPLC, TCA (12%) was added directly to the bacterial suspension.

For growth in the absence of oxygen, the bacteria were incubated in sterile tubes with screw caps (Greiner Bio-One BVBA/SPRL, Wemmel, Belgium). The culture was sparged with N_2 _for 1 min and the tubes were hermetically closed before incubation.

### Determination of thiamine compounds and adenine nucleotides

Thiamine compounds were determined by HPLC as previously described, after conversion to fluorescent thiochromes [29] and ATP was determined by luciferin luminescence using the Bac-Titer-Glo kits (Promega Benelux b.v., Leiden, The Netherlands). For determination of the energy charge [20], ATP, ADP and AMP concentrations were determined by a HPLC method, using fluorescence detection after ethenylation with chloroacetaldehyde [30]. Intracellular concentrations were estimated assuming an intracellular volume of 3.2 μL per mg of protein [8].

### Determination of oxygen consumption

O_2 _consumption was determined polarographically using a Clark-type electrode (Hansatech, King's Lynn, Norfolk, UK) in a 2 mL cell at 25 or 37°C in M9 minimal medium. When a linear basal O_2 _consumption was reached, either (10 mM) D-glucose, L-lactate or L-malate was added, followed by KCN (1 mM) or CCCP (0.1 - 50 μM).

### Separation of free and bound ThDP and AThTP using a molecular sieve

BL21 bacteria grown overnight in LB medium were transferred to M9 medium without glucose. After incubation for 4 h (37°C, 250 rpm), the samples were sonicated (100 kHz, 3 × 30 s with 1 min intervals) on ice and centrifuged (5 min, 10,000 × g, 4°C). The supernatant was injected (100 μL) on a TSK column (G3000SW, 30 × 0.75 cm, 10 μm, Tosoh, Bioscience GmbH, 70567, Stuttgart, Germany) equilibrated in Na acetate buffer (25 mM, pH 7.2) at a flow rate of 0.5 mL/min. Fractions of 1 mL were collected and thiamine derivatives were determined after treatment with TCA as described above.

## List of abbreviations

AK: adenylate kinase; AThTP: adenosine thiamine triphosphate; CCCP: carbonyl cyanide 3-chlorophenylhydrazone; GST: glutathione S-transferase; hThTPase: recombinant human thiamine triphosphatase; IPTG: isopropyl β-D-1-thiogalactopyranoside; P_i_: inorganic phosphate; (p)ppGpp: guanosine 3',5' tetra- and pentaphosphate; TCA: trichloroacetic acid; ThDP: thiamine diphosphate; ThMP: thiamine monophosphate; ThTP: thiamine triphosphate; ThTPase: thiamine triphosphatase; Δp: proton-motive force.

## Authors' contributions

TG made most of the experimental work. BL and PW participated in the design of the study and the interpretation of the data. BEM and WZ contributed to the interpretation of the data and were responsible for the respiratory experiments. LB was the project leader. The manuscript was written by LB and PW. All authors read and approved the study.
